# Flexible Titanium Intramedullary Nail Displacement After Magnetic Resonance Imaging

**DOI:** 10.5435/JAAOSGlobal-D-23-00004

**Published:** 2023-09-22

**Authors:** Katherine Velicki, Julianna Mazziotti, Connor Pihl, Scott Yang

**Affiliations:** From the Department of Orthopaedic Surgery, Oregon Health & Science University, Portland, OR.

## Abstract

**Case:**

A previously healthy 7-year-old boy presented with midshaft radius and ulna malunion after 8 weeks of nonsurgical treatment. He underwent open reduction and internal fixation of both bones with titanium alloy nails and was placed in a long arm cast. Four weeks after surgery, the patient underwent sedated brain MRI and woke up from anesthesia with elbow pain. On cast removal, the ulnar flexible nail was noted to have displaced proximally by 1.5 cm.

**Conclusion:**

Despite meeting American Society for Testing and Materials standards, untethered titanium orthopaedic implants are at risk of clinically significant displacement in the early postoperative period.

Most orthopaedic surgeons are familiar with the dangers of metallic objects in the presence of a magnetic resonance imaging (MRI) field. Alterations in proton rotation during an MRI scan can cause metallic implants to loosen, heat, or damage nearby tissues. As more patients undergo advanced imaging in the United States each year, it is important that clinicians are aware of the rare but important adverse events associated with these tests.^1^

The literature contains many studies that examine the safety of orthopaedic implants in MRI fields. However, the majority are ex vivo laboratory studies of implant properties, such as torque, translational force, or heating.^2^ This case report describes the translation of an ulnar flexible intramedullary nail after a 7-year-old boy underwent a brain MRI with sedation in the early postoperative period.

## Statement of Informed Consent

The patient's parents consented to have his clinical experience submitted for publication in a medical journal.

## Case Report

**Figure 1:**
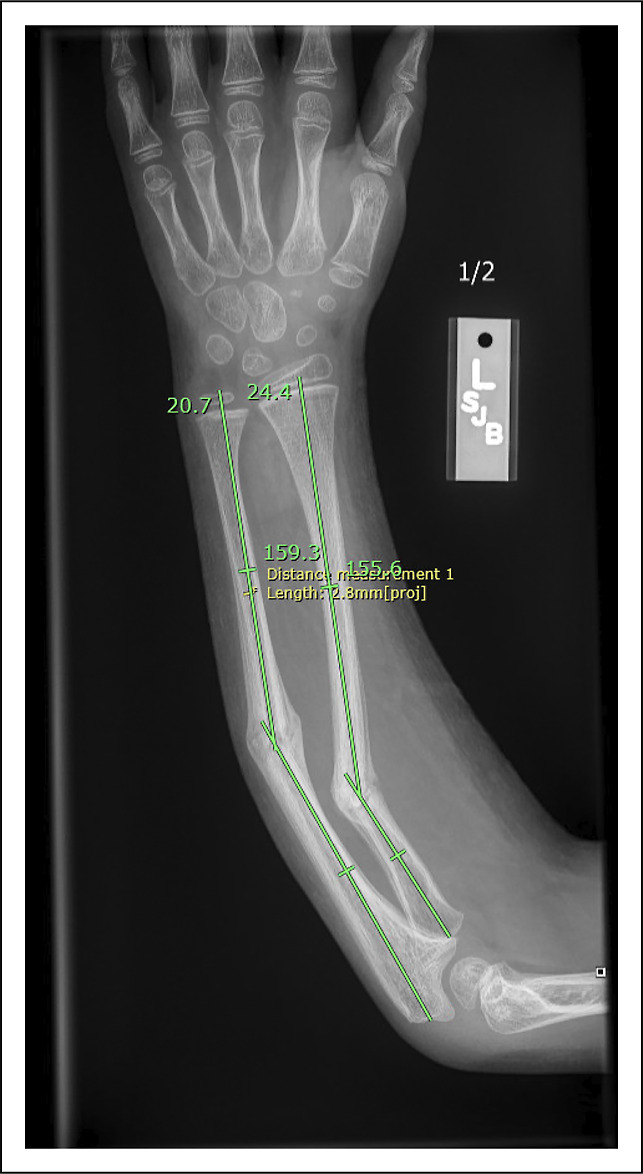
Radiograph showing radial and ulnar diaphyseal malunion after 8 weeks of closed treatment in a long arm fiberglass cast.

A previously healthy 7-year-old boy presented to a specialty pediatric orthopaedic regional referral center for evaluation of left midshaft radius and ulna fracture malunion (Figure 1). The patient sustained the injury from a ground-level fall 8 weeks before presentation, with initial nonsurgical treatment (closed reduction and long arm casting) managed by a local orthopaedic surgeon. On clinical examination, the patient had no tenderness at fracture sites and had limited pronation and supination with a firm mechanical block with an approximately 30° arc of motion. Radiographs demonstrated healing transverse diaphyseal fractures, with approximately 30° and 20° of apex ulnar angulation in the radius and ulna, respectively (Figure 1). Surgical intervention, including malunion takedown and open reduction and internal fixation of the left distal radius and ulna with intramedullary fixation, was recommended to address the unacceptable deformity and secondary pronosupination range-of-motion deficit.

**Figure 2:**
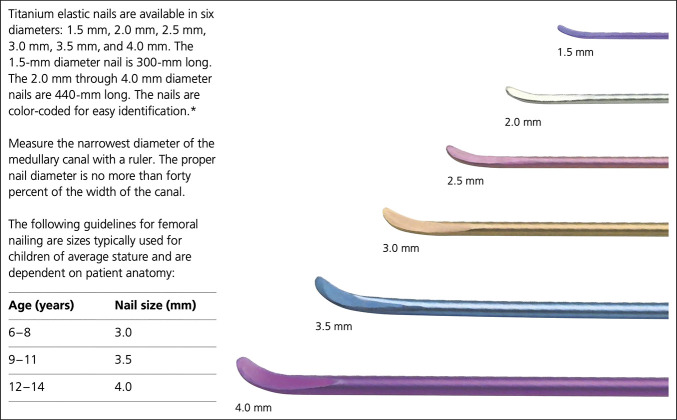
Illustration showing an example of a 2-mm titanium alloy elastic nail similar to that used in this case.

**Figure 3:**
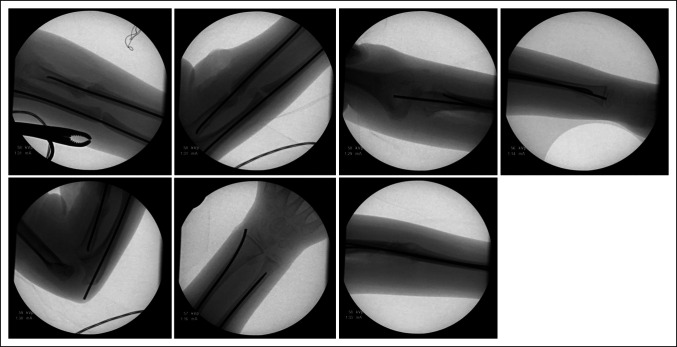
Intraoperative fluoroscopy images showing the elastic nail position at the end of malunion takedown and open reduction and internal fixation.

**Figure 4:**
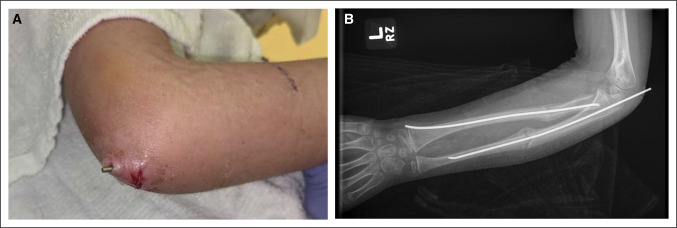
Clinical photograph (**A**) and anteroposterior radiograph (**B**) showing the nail position at the 4-week follow-up appointment.

**Figure 5:**
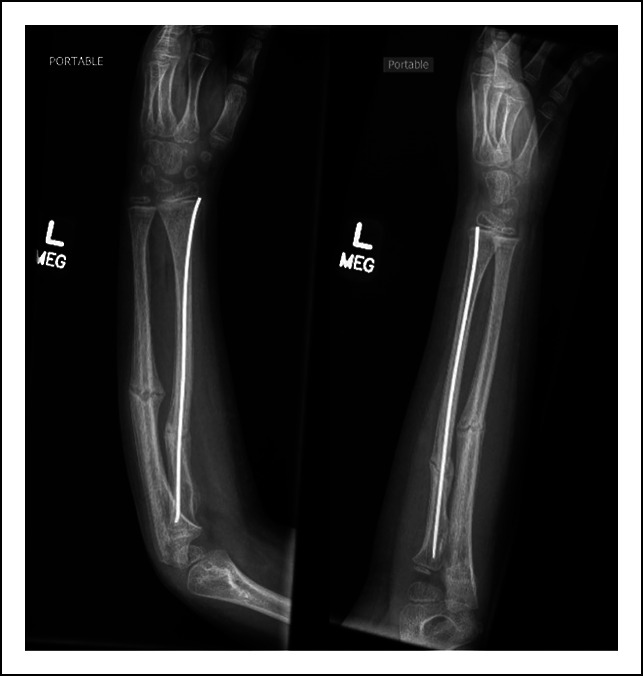
Radiographs (anteroposterior and lateral views) at the 2-week follow-up appointment after ulnar rod removal.

**Figure 6:**
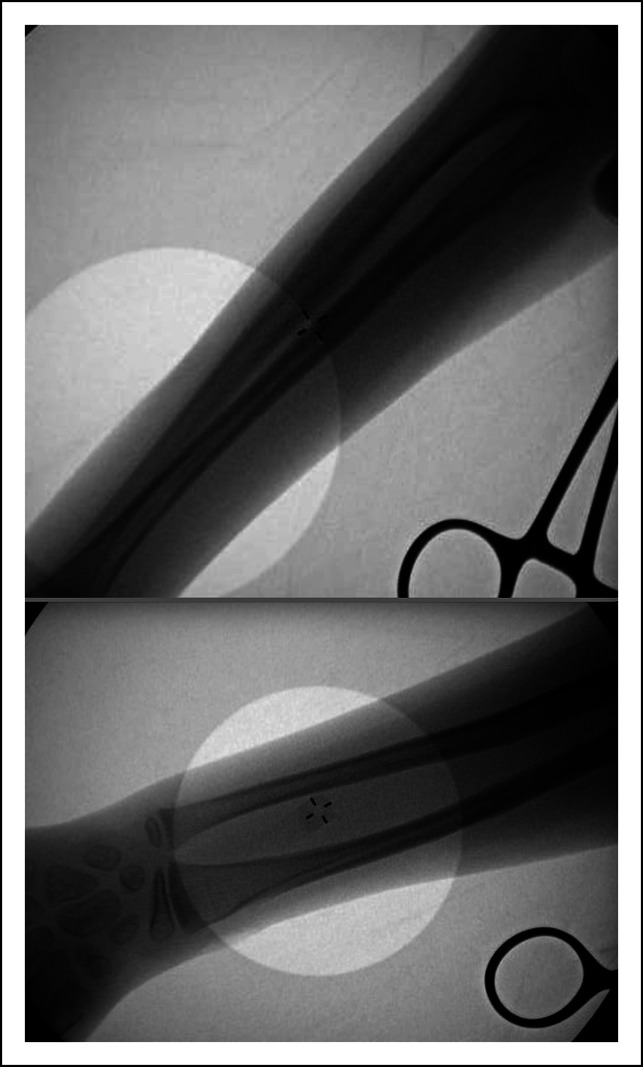
Flouroscopic images at the time of hardware removal (1 year post-injury).

In the operating room, closed manipulation of the fractures was first attempted. The ulna was acceptably reduced and stabilized with an antegrade 2.0-mm titanium elastic nail (Figure 2). Open treatment of the proximal radius was required. A standard volar approach was used over the radius malunion to allow for open takedown and anatomic reduction with clamps. A retrograde 2.0-mm titanium elastic nail was then placed using a mini radial styloid approach. Postoperative fluoroscopic images demonstrated excellent implant placement and stable fracture reduction with dynamic examination (Figure 3). Rods were cut and tamped to appropriate depth, and a long arm fiberglass cast was placed. The patient was discharged home after PACU recovery with plans for clinic follow-up in 5 weeks for cast removal and repeat radiographs. Four weeks after surgery, the patient had a syncopal event without a fall and was admitted to an outside hospital for expedited workup. He underwent a sedated brain MRI with and without contrast that revealed a Chiari malformation. Immediately after MRI, the patient reported new left elbow pain that was not present before the MRI. He was seen in pediatric orthopaedic clinic 4 days later. On cast removal, the ulnar nail was noted to have displaced proximally by approximately 1.5 cm, tunneling through the patient's skin (Figure 4). The patient was returned to the operating room later that day for removal of the ulnar intramedullary nail, irrigation and débridement of the left elbow wound, and repeat long arm casting. The patient was discharged home after PACU recovery with 1 week of oral antibiotic prophylaxis and plans for clinic follow-up in 2 weeks for cast removal and wound check and radiographs (Figure 5). The patient's forearm fractures healed uneventfully, without wound complications or malunion. His radius intramedullary nail was removed electively at approximately 1 year postoperatively (Figure 6). He regained full forearm pronosupination arc of motion that was symmetric to his contralateral forearm.

## Discussion

Considering the timing of events, the authors suspected that this patient's brain MRI caused the ulnar nail to displace. According to the manufacturer, nonclinical testing of the titanium elastic nail did not reveal any relevant torque or displacement of the construct in a 3-Tesla (T) MRI field. In other words, it is unlikely that a rapid translational force propelled the nail through the patient's skin. However, it is possible that the MRI produced vibrations in the nail that gradually shifted its position over the course of this study.

The displaced elastic nail is an alloy composed of titanium, aluminum, and niobium.^3^ Titanium is a paramagnetic metal that is only weakly magnetized by an external magnetic field and is generally considered to be MRI-compatible.^4^ Nonclinical testing for this particular implant under a 3-T magnetic field revealed torque and displacement within ASTM standards. However, individual implants are not tested before placement in patients, and MR safety testing relies on laboratory-based experiments that fail to simulate the complex spatial relationships between implants, human tissues, and transmission coils that occur in the clinical environment. For these reasons, it is important that patients with MR-compatible implants still be monitored carefully for temperature and pain sensations during their scans.

The orthopaedic literature contains many studies of titanium implant torque, displacement, and temperature change under various MR field strengths. Several have reported notable titanium implant displacement under a 7-T magnetic field. For instance, Feng et al.^5^ measured a 44° deflection angle in a titanium proximal femur plate, just 1° short of exceeding the force of gravity and failing to meet ASTM standards. They also reported mild torque values in two titanium-based hip implants, meaning that the device slightly changed its orientation during exposure to the 7-T field. Another study by Dula and colleagues^6^ measured a 45° deflection angle and moderate torque in a titanium alloy hip stem.

The literature also contains numerous reports supporting the safety of titanium-based orthopaedic implants during MRI. Titanium alloy plates and screws tested by Zou et al.^7^ showed an average deflection angle of only 4.3° under a 1.5-T field. Titanium and titanium alloy spinal rods deflected by less than 3° in a 3-T field in a report by Tsukimura et al.^8^ Only two retrospective studies have assessed for MRI-induced orthopaedic implant migration in live patients. Neither reported any issues with implant migration, loosening, nonunion, or compromised fixation.^9,10^

In general, the risk of orthopaedic implant-based complications related to MRI is extremely low. Most clinically used MRI scanners in the United States produce fields less than 3.0 T, and displacement forces on orthopaedic implants, especially, are almost universally overcome by rigid fixation to bone (eg, screws or interlock devices). This case report presents a unique scenario in which the orthopaedic implant was not well-fixed to bone. In addition, our sedated pediatric patient was unable to report pain from implant displacement until waking from anesthesia.

The major weakness of this case report is that one cannot prove that activities of daily living did not contribute to rod migration. The authors think that this is unlikely given the time course of the patient's symptoms after his MRI, the amount of rod migration, and that the arm was immobilized in a well-molded long arm cast.

This experience suggests that regardless of meeting ASTM standards, untethered titanium orthopaedic implants are at potential risk of clinically significant displacement in an MR field, and special consideration should be given to patients who cannot communicate their symptoms during MRI.
